# *In Vivo* Study of the Effects of ER*β* on Apoptosis and Proliferation of Hormone-Independent Prostate Cancer Cell Lines PC-3M

**DOI:** 10.1155/2018/1439712

**Published:** 2018-06-19

**Authors:** Changli Zhou, Chunyu Yu, Lirong Guo, Xige Wang, Huimin Li, Qinqin Cao, Feng Li

**Affiliations:** ^1^School of Nursing, Jilin University, 965 Xinjiang Street, Changchun, Jilin 130020, China; ^2^Basic Medical School, Jilin University, 126 Xinmin Street, Changchun, Jilin 130020, China

## Abstract

**Objective:**

To evaluate the* in vivo* therapeutic effects of attenuated Salmonella carrying PCDNA3.1-ER*β* plasmid in hormone-independent prostatic cancer in nude mice and to clarify the mechanism by which estrogen receptor *β* (ER*β*) induces apoptosis and proliferation in prostatic cancer cells in mice.

**Methods:**

The orthotopic prostatic cancer models of mice were randomly divided as follows: MOCK group, treated with PBS, PQ group, treated with attenuated Salmonella alone, PQ-PCDNA3.1 group, treated with attenuated Salmonella carrying PCDNA3.1 plasmid, and PQ-PCDNA3.1-ER*β* group, treated with the attenuated Salmonella carrying PCDNA3.1-ER*β* plasmid. Then, 10 *μ*l of the plasmid-containing solution, comprising 1 × 10^7^ cfu of the bacteria, was administered via intranasal delivery to each group except the MOCK group. The experimental methods included flow cytometry and terminal deoxyribonucleotidyl transferase-mediated dUTP-digoxigenin nick end-labelling (TUNEL) assay, immunohistochemistry, and western blotting.

**Results:**

Compared with the MOCK, PQ, and PQ-PCDNA3.1 groups, the weights of tumors in the PQ-PCDNA3.1-ER*β* group were significantly reduced. The results of flow cytometry and TUNEL assay revealed that the number of apoptotic cells in the PQ-PCDNA3.1-ER*β* group significantly increased. Compared with PQ-PCDNA3.1 group, the protein expression levels of ER*β*, Bad, p-caspase 9, p-caspase 3, and cleaved PARP in the PQ-PCDNA3.1-ER*β* group were significantly increased, while the expression levels of Akt, p-Akt, and Bcl-xl were decreased (*P* < 0.05).

**Conclusion:**

The attenuated Salmonella carrying PCDNA3.1-ER*β* plasmid could inhibit the growth of orthotopic prostatic cancer in mice by increasing the expression of ER*β*.

## 1. Introduction

Estrogen is an important hormone in humans. Studies have shown that estrogen signaling plays a significant role in the normal and abnormal growth of the prostate gland [1, 2]. In 1996, the discovery of estrogen receptor beta (ER*β*) in rats [3] and humans [4] changed our understanding of the estrogen signaling. The effects of estrogen on target tissues are now known to be mediated by estrogen receptor alpha (ER*α*) and ER*β*, which are members of the nuclear hormone receptor family and ligand-activated transcription factors. Expression of estrogen receptors has been found in many other tumors such as breast, uterus, ovarian, colon, and prostate and also identified in bladder, lung cancer, and so on [5, 6]. The human prostate is equipped with a dual system of ERs: ER*α* and ER*β*, and they undergo significant remodeling in the process of prostate cancer (PCa) development and progression [7–9]. ER*β* is expressed at high levels in the normal prostate, mostly localized to both basal and luminal of the normal prostate [7, 10]. However, there is growing evidence showing that ER*β* is gradually lost in cancer progression.

In the studies of Asgari and Morakabati, it was shown that ER*β* expression is significantly lower in high grade tumors than in low or intermediate-grade tumor [11]. Leav et al. showed that ER*β* staining greatly diminished in most cases of grade 4/5 PCa [8]. Horvath et al. using different primary antibodies studied ER*β* expression patterns in normal, hyperplastic, and prostate cancer; they found that ER*β* is highly expressed in normal human prostate, majority in the basal compartments of the epithelium, while more than 75% of PCs did not express ER_*β*_. Additionally, there was a progressive loss of its expression in invasive PCa [12]. Fixemer et al. suggested that ER*β* protein expression decreased during PCa progression [9]. Latil et al. also have shown a decreased expression of ER*β* in prostate carcinoma when compared to nonpathological tissues, and the loss of ER_*β*_ expression is associated with a higher Gleason grade and higher metastatic potential [13]. These studies about loss of ER_*β*_ expressions during carcinogenesis add to an accumulating body of evidences supporting a protective role of ER_*β*_. Pasquali et al. [14] hypothesized that the loss of ER_*β*_ may promote cell proliferation and, possibly, carcinogenesis by some unknown mechanism based on the loss of ER_*β*_ expression in prostate hyperplasia and carcinoma. Chang and Prins, Poelzl et al., and Signoretti and Loda suggested that ER_*β*_ might exert a protective effect against aberrant cell proliferation and/or carcinogenesis [1, 15, 16]. Weihua et al. proposed that ER_*β*_ has antiproliferation and proapoptotic function in the prostate [17]. Furthermore, findings in ER_*β*_ knockout mice indicated that these animals develop prostatic hyperplasia at an old age, a phenomenon that does not occur in ER*α*knockout mice [18]. This evidence suggests the potential protective role of ER_*β*_ in potent protective role in prostate epithelial cells. Moreover, studies by McPherson et al. and Imamov et al. have shown the antiproliferative activity of ER_*β*_ agonists in the prostate [19, 20]. Additionally, a number of studies have also identified novel therapeutic agents that target ER_*β*_ in PCa and induce apoptosis in prostate cell lines [21, 22]. Based on the current knowledge about ER*β* and our previous studies, recombination plasmid PCDNA3.1-ER*β*, which contains the human estrogen receptor 2, ESR2 (ER*β*), full-length cDNA was constructed to increase the ER*β* expression. Because the effects of ER*β* in transfected PC-3M cells and the fact that ER*β* can inhibit the cells' proliferation and induce apoptosis are already known, the primary objective of this study is to observe the* in vivo* therapeutic effect of attenuated Salmonella carrying PCDNA3.1-ER*β* plasmid in hormone-independent PCa in nude mice and clarify the mechanism by which ER*β* induces apoptosis in PCa cells.

## 2. Materials and Methods

### 2.1. PQ-PCDNA3.1-ER*β*Plasmid and Bacteria

The attenuated Salmonella phoP/phoQ strains and PQ-PCDNA3.1 plasmids were available in our laboratory. The ER*β* gene with the GenBank accession number NM-001437 was used in the present study. PCDNA3.1-ER*β* plasmid was constructed in our laboratory. The attenuated Salmonella phoP/phoQ strain was used as the vector to carry the PCDNA3.1-ER*β* plasmid. The PCDNA3.1-ER*β* plasmid was then transduced into the attenuated Salmonella phoP/phoQ strain by electroporation (2.5 kV, 25 mF, 200 Ω, pulse time 0.03 s) [23]. The plasmid in the Salmonella transfectant was extracted to verify the successful transfection. The product was then subjected to agarose electrophoresis for visualization.

### 2.2. Cell Culture and Establishment of Mouse Orthotopic Prostate Cancer Models

The human prostate cancer cell line PC-3M was available at our laboratory. These cells were grown in Iscove's modified Dulbecco's medium (GIBCO, Carlsbad, CA, USA) containing 10% fetal bovine serum (GIBCO). Then PC-3M cells (1.5 × 10^6^ cells per 100 *μ*l) were transplanted into four mice subcutaneously to generate primary cancer. 4–6-week-old male BALB/C nu/nu mice, weighing 18~22 g, were purchased from the Beijing Institute for Experimental Animals (Beijing, China). All animals were housed and experiments were performed according to the guidelines established by Jilin University for the ethical use of animals in research. Then, the tumor growth status was observed every alternate day. After the development of a palpable tumor at the site of inoculation, the tumors were excised and placed in the Hypothermia Sterile Saline. Suitable sections of the tumor tissue were cut into 1.5 mm^3^ blocks and implanted by surgical orthotopic implantation between two lobes of the prostatic gland in a recipient group of BALB/C nu/nu mice, according to methods described previously [24]. Three days after implantation, the mice that survived operation were randomly divided into four groups (*n* = 8 per group): (i) MOCK group, which was given PBS as PBS control; (ii) PQ group, which was given attenuated Salmonella alone as attenuated Salmonella control; (iii) PQ-PCDNA3.1 group, which was given attenuated Salmonella carrying PCDNA3.1 empty plasmid as empty plasmid control; (iv) PQ-PCDNA3.1-ER*β* group, which was given attenuated Salmonella carrying PCDNA3.1-ER*β* plasmid as experiment group. The bacteria were grown overnight on LB medium and then diluted by 1 : 100 in LB medium. Bacteria were harvested at the late-log phase, washed, and diluted in PBS. Then, 10 *μ*l of PBS (pH 7.6) was administered to the mice in PBS control group, and 1 × 10^7^ colony forming units (cfu) of attenuated Salmonella carrying different plasmids were administered to the mice in the attenuated Salmonella control, empty control, and experiment groups by intranasal (i.n.) delivery. The mice were anesthetized by intraperitoneal injection with 0.1 ml of 1% pentobarbital sodium and administered 10^7^ cfu of attenuated Salmonella [25]. This process was repeated on day 10. Mice were sacrificed on day 32, and the tumors harvested from the different groups were weighed and processed for Annexin V-FITC staining, immunochemistry, terminal deoxyribonucleotidyl transferase-mediated dUTP-digoxigenin nick end-labelling (TUNEL) assays, and western blotting.

### 2.3. Annexin V and Propidium Iodide (PI) Staining

Apoptosis was detected by flow cytometry using Annexin V-FITC/PI Apoptosis Detection Kit according to the manufacturer's instructions (Nanjing, KeyGen Biotech, Nanjing. China). The tumor cells were collected, washed twice with PBS, and then resuspended in 500 *μ*l of staining solution containing FITC-conjugated Annexin V antibody (5 *μ*l) and propidium iodide (PI). After incubation on ice for 30 min, cells were analyzed by flow cytometry. Early apoptosis is defined by Annexin V+/PI− (Q4) and late apoptosis is defined by Annexin V+/PI+ staining (Q2) as determined by FACS can (Beckman Coulter cell, CA, USA).

### 2.4. Immunohistochemistry and TUNEL Assay

Serial sections of the tumor tissue obtained from mice were fixed in formalin. Immunostaining was performed using the Vectastain Elite ABC avidin/biotin staining kit (Vector Laboratories Inc., Burlingame, CA, USA). Antibodies specific to proliferating cell nuclear antigen (PCNA) were purchased from Santa Cruz Biotech, Inc (China, Asia). The DeadEnd Fluorometric TUNEL System (Promega) was used to measure the fragmented DNA in apoptotic cells by catalytically incorporating fluorescein-12-dUTP at the 3′-OH DNA ends using recombinant terminal deoxynucleotidyl transferase (rTdT) (Promega). Paraffin-embedded tissues were cut into 3-*μ*m sections, deparaffinized, and hydrated according to standard protocol [26]. After incubation with proteinase K (20 *μ*g ml^−1^) for 30 min at room temperature, the TUNEL reaction mix containing rTdT and the rTdT reaction mix were added to the slides, followed by incubation in a humidified chamber for 60 s at 37°C. After being washed, the sections were immersed in 40 ml of freshly prepared propidium iodide solution (1 *μ*g ml^−1^) for 15 min at room temperature in the dark. The staining was visualized by a laser scanning confocal microscope. TUNEL-positive cells exhibited green fluorescence.

### 2.5. Western Blot Analysis

For western blot analysis, lysate proteins (45 *μ*g) were separated by 12% or 15% w/v SDS-polyacrylamide gel electrophoresis. The separated proteins were then transferred onto nitrocellulose transfer membranes (0.2 or 0.45 *μ*m, Millipore, Bedford, MA). The membranes were blocked with 5% nonfat dry-milk in a buffer (10 mM Tris-HCL [pH 7.6], 100 mM NaCl, and 0.1% Tween 20) for 1 h at room temperature, incubated with the desired primary antibodies overnight at 4°C and then incubated with alkaline phosphatase-conjugated goat anti-rabbit secondary antibodies at a 1 : 1000 dilution for 1 h at room temperature as previously described [27]. After washing, the proteins were detected using Odyssey Infrared Imaging System (LI-COR Biosciences, Lincoln, NE). Protein levels were quantified by densitometry using Quantity One software (Bio-Rad). Antibodies against *β*-actin, ER*β*, Bad, and Bcl-xl were obtained from Cell Signaling Technology, and antibodies against Akt, p-Akt (Ser473), p-caspase 9, p-caspase 3, and cleaved PARP were obtained from Santa Cruz Biotech, Inc.

### 2.6. Data Analyses

Quantitative data were expressed as mean ± standard error (SE). The significance was determined using the *t*-test. *P* < 0.05 was deemed statistically significant.

## 3. Results

### 3.1. Antitumor Activity of ER*β*

To evaluate the effects of PCDNA3.1-ER*β* plasmid on the growth of prostate cancer* in vivo*, the orthotopic prostatic cancer models of the mice were developed to determine the antitumor efficacy. Three days after operation, cancer-bearing mice were intranasally given either PBS, attenuated Salmonella alone, attenuated Salmonella carrying PCDNA3.1 plasmid, or attenuated Salmonella carrying PCDNA3.1-ER*β* plasmid, which was repeated on day 10. The animals were sacrificed on day 32, and the tumor weights were determined (Table 1). Compared to the PQ-PCDNA3.1-ER*β* group, in the mice from the MOCK, PQ, and PQ-PCDNA3.1 groups, the degree of cachexia was notable. A significant difference was observed between the mean body weight and mean tumor weight of the mice in the MOCK, PQ, and PQ-PCDNA3.1 groups and that of mice in the PQ-PCDNA3.1-ER*β* group (*P* < 0.05).

### 3.2. Assessment of Apoptosis by Annexin V-FITC Staining

**Figure 1:**
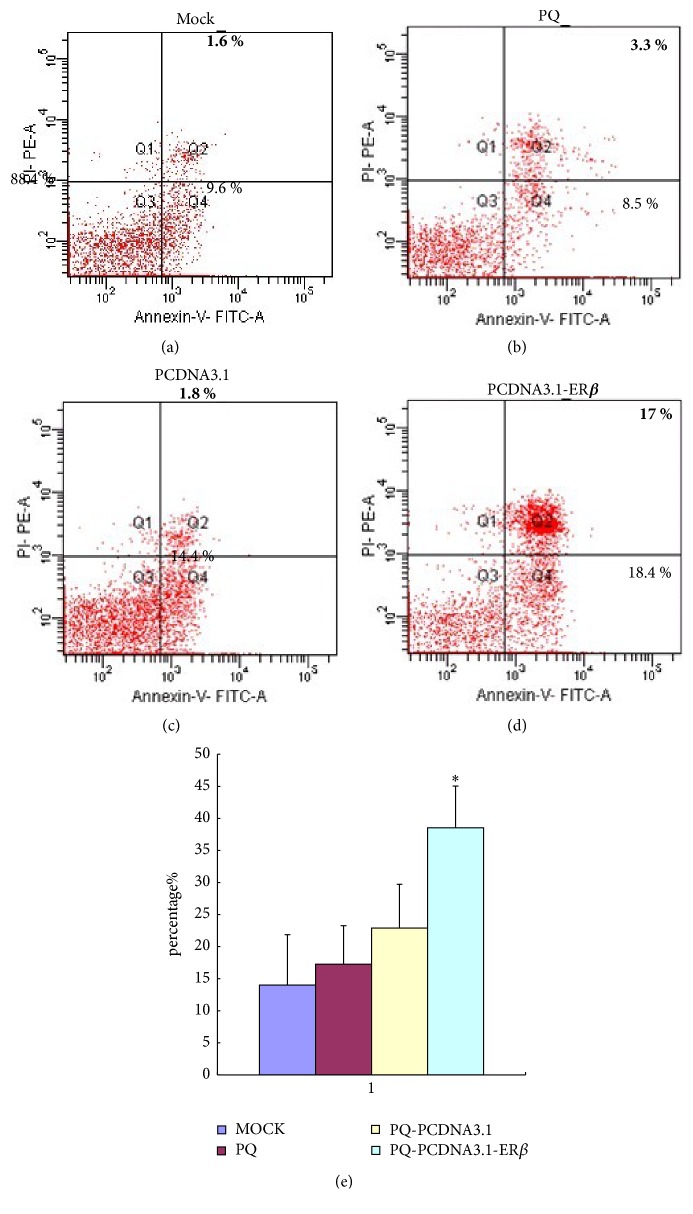
Assessment of apoptosis by Annexin V/PI staining of prostate cancer tissues. (a) MOCK group; (b) PQ group; (c) PQ-PCDNA3.1 group; (d) PQ-PCDNA3.1-ER*β* group; (e) percentage of cell death based on the assessment of apoptosis by Annexin V/PI staining. *∗* indicates that apoptosis induced by PQ-PCDNA3.1-ER*β* was significantly greater than that in the MOCK, PQ, and PQ-PCDNA3.1 groups (*P* < 0.05).

30 mg of the fresh tumor tissues was weighed immediately after the mice were sacrificed; the tumor tissues were then crushed and apoptosis was examined by flow cytometry (Figure 1). Quantitative analysis using the Annexin V/PI assay showed that, in the PCDNA3.1-ER*β* group, the proportion of early-stage apoptotic cells (Annexin V+/PI−) increased significantly to 18.4%, and the proportion of late-stage apoptotic cells (Annexin V+/PI+) increased significantly to 17% (Figure 1(d)). Apoptosis induced by PQ-PCDNA3.1-ER*β* was significantly greater than that in the MOCK, PQ, and PQ-PCDNA3.1 groups (*P* < 0.05) (Figure 1(e)).

The positively stained cells were counted using FACScan. Data were presented as the mean ± SE, *n* = 3. *∗* indicates that *P* < 0.05, compared to the MOCK, PQ, and PQ-PCDNA3.1 groups.

### 3.3. TUNEL and PCNA Assays

**Figure 2:**
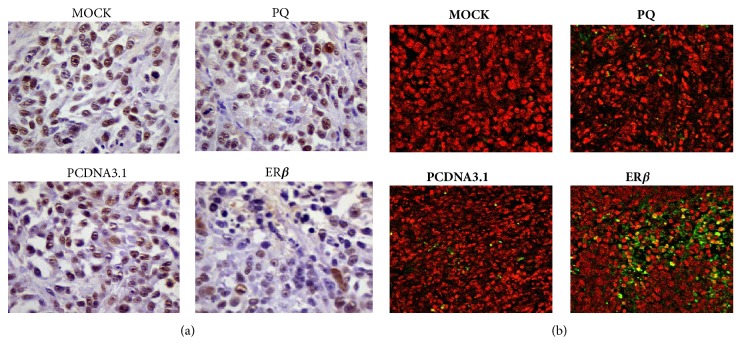
PCNA and TUNEL assay in tumor tissues. (a) Immunohistochemistry was performed in tumor tissues from each group to measure cell proliferation by PCNA staining. (b) TUNEL assay in prostate tumors from each group.

Tumor cell proliferation and apoptosis can regulate the tumor size at any given time-point. Therefore, we performed immunohistochemistry on the tumor tissues to measure the proliferation expression of PCNA (Figure 2(a)) and TUNEL (Figure 2(b)) assay to measure apoptosis. Immunohistochemistry was performed in tumor tissues derived from each group to measure cell proliferation by PCNA staining. The brown granules in the nuclei indicate the positive proliferating cells. Compared to the MOCK group, PQ, PQ-PCDNA3.1, and PQ-PCDNA3.1-ER*β* group, the PCNA protein expression decreased. The percentage of apoptosis was measured on histologic sections of tumors using the TUNEL assay. Tumors from each group of mice treated with PBS, PQ, PQ-PCDNA3.1, and PQ-PCDNA3.1-ER*β* were evaluated. Green fluorescence represents apoptotic cells. Relatively few apoptotic cells were detected in the tumors from the MOCK, PQ, and PQ-PCDNA3.1 groups, but a comparatively larger number of apoptotic cells were present in the tumors from the PQ-PCDNA3.1-ER*β* group.

### 3.4. Expression Levels of ER*β* and Apoptosis-Associated Genes

**Figure 3:**
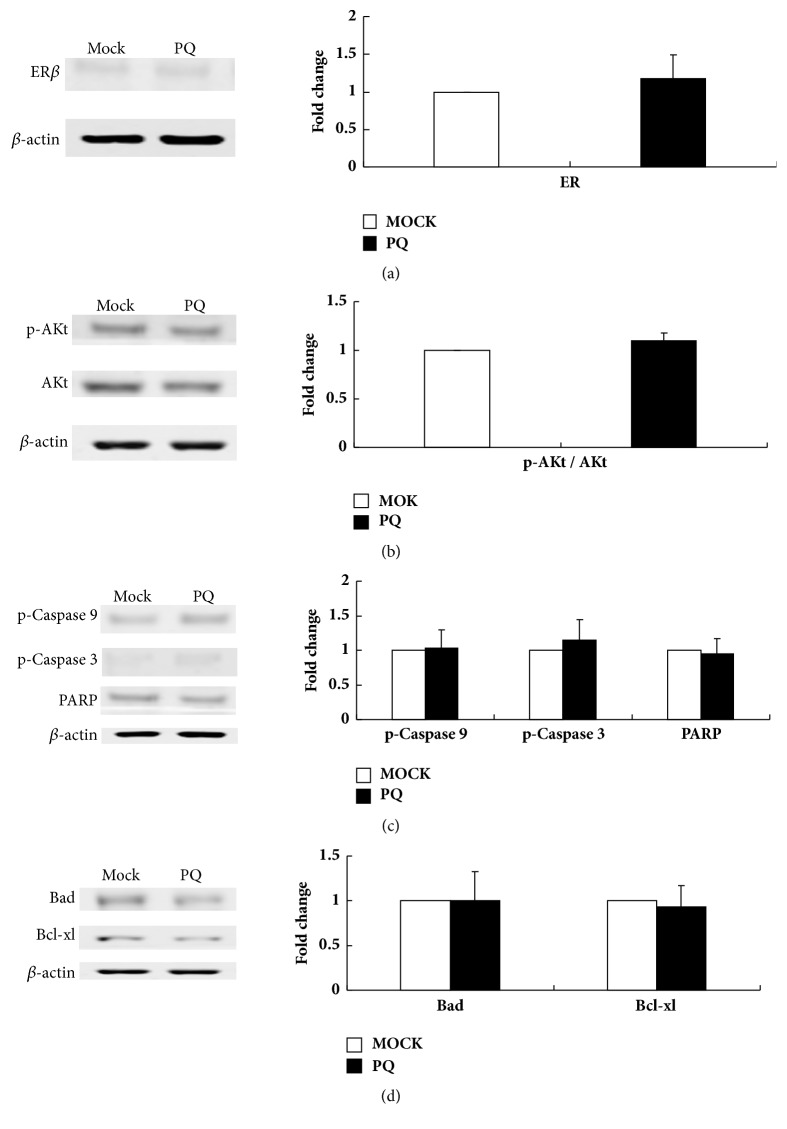
Representative photographs from western blot assay of tumor tissues from the MOCK and PQ groups and the quantification of these genes at the protein level. (a) The expression of ER; (b) the expression of Akt and p-Akt; (c) the expression of p-caspase 9, p-caspase 3, and PARP; (d) the expression of Bad and Bcl-xl.

**Figure 4:**
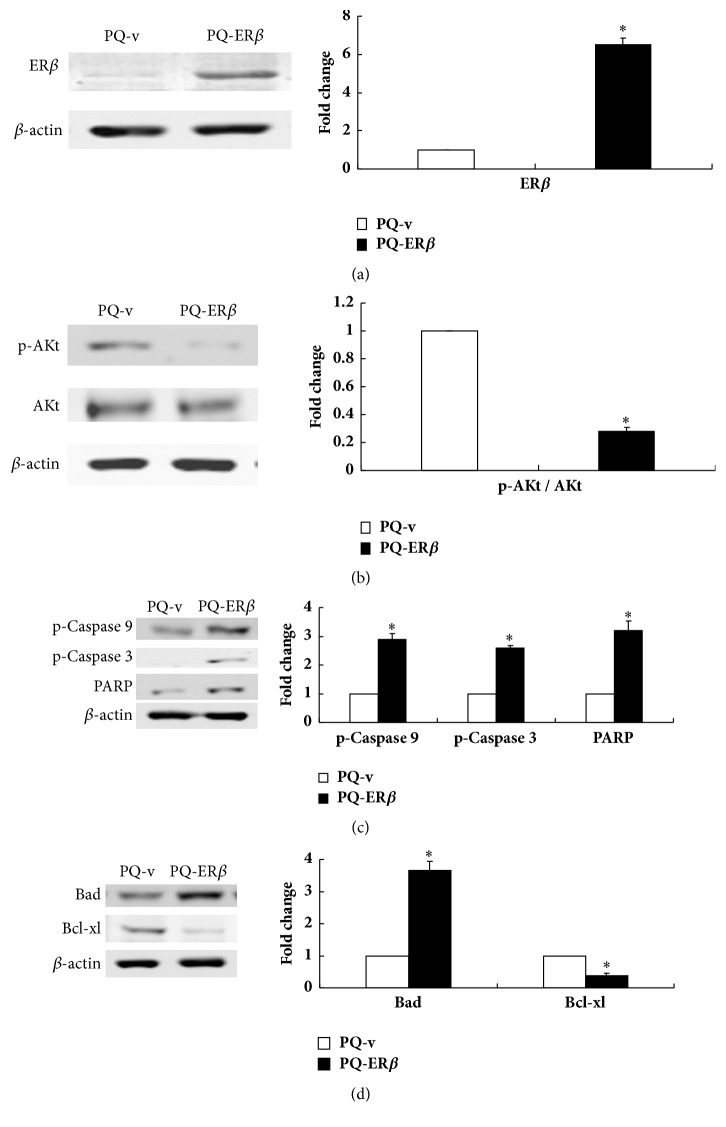
Representative photographs from western blot assay of tumor tissues from the PQ-v and PQ- ER*β* groups, and quantification of these genes at the protein level. (a) The expression of ER; (b) the expression of Akt and p-Akt; (c) the expression of p-caspase 9, p-caspase 3, and PARP; (d) the expression of Bad and Bcl-xl in the PQ-v and PQ-ER*β* groups. PQ-v: PQ-PCDNA3.1 group; PQ-ER*β*: PQ-PCDNA3.1-ER*β* group. *∗* indicates that apoptosis induced by PQ-PCDNA3.1-ER*β* was significantly greater than that in the MOCK, PQ, and PQ-PCDNA3.1 groups (*P* < 0.05).

To further determine the effects of PCDNA3.1-ER*β* treatment on the expression of ER*β* and apoptosis-associated genes, a western blot was performed. First, we compared the results of the MOCK and PQ groups to study the effects of the attenuated Salmonella on the tumors. The results showed that no significant changes were observed in the levels of ER*β*, Akt, p-Akt, Bad, Bcl-xl, p-caspase 9, p-caspase 3, and cleaved PARP (Figure 3). We then compared the expression levels of these genes in the PQ-PCDNA3.1 and PQ-PCDNA3.1-ER*β* groups; the results showed that the levels of ER*β*, Bad, p-caspase 9, p-caspase 3, and cleaved PARP proteins were significantly elevated in tumors, but the levels of Akt, p-Akt, and Bcl-xl levels were downregulated after PQ-PCDNA3.1-ER*β* treatment (Figure 4). These results imply that PQ-PCDNA3.1-ER*β* treatment could promote the apoptosis of tumor cells.

## 4. Discussion

PCa is the most common malignant tumor in the elderly man. Its incidence differs among countries and ethnic groups [28]. The etiology of PCa seems to be multifactorial, influenced by factors such as diet, race, and alteration of genes and hormones [29, 30]. The standard therapy for prostate cancer is surgery, radiotherapy, and androgen deprivation therapy [31]. Unfortunately, the tumor inevitably transforms into an androgen independent state and proceeds to develop further. Thus, the need of the hour is the development of newer and more effective strategies to treat PCa.

Although the precise biological function of ER*β* is not completely defined, it has been suggested that it may protect the normal prostate epithelium from undergoing unscheduled cell proliferation by acting via binding to estrogen [8, 32]. The studies by Horvath et al. [33] and Leav et al. [8] demonstrated the reduction in ER*β* expression during carcinogenesis, suggesting that ER*β* might be important for the maintenance of normal prostate epithelium. Ricke et al. and van Agthoven et al., after studies on prostate cancer cell lines, pointed out that ER*β* activation could induce apoptosis and decrease cell proliferation [34, 35]. Moreover, in some* in vitro* settings, ER*β* inhibits the proliferation, migration, and invasion of breast cancer cells [36, 37]. For these reasons, ER*β* could be used as a potential target in antitumor therapy [38].

In recent years, the use of attenuated Salmonella for cancer therapy research continues to increase. Low et al. [39] and Pawelek et al. [40] reported that tumor-targeted Salmonella exhibited tumor accumulation ratios in excess of 1000 : 1 compared with normal tissues. They are highly invasive and have a low pathogenicity and can be administered via oral, intraperitoneal, intravenous, and intranasal means [41–44]. Zhang et al. [45] have studied the effect of attenuated Salmonella as a carrier for the si-RNA-Stat3 plasmid to treat PCa. The* Salmonella enterica* serovar typhimurium* (S. typhimurium)* phoP/phoQ operon is composed of a membrane-associated sensor kinase (PhoQ) and a cytoplasmic transcriptional regulator (PhoP); phoP/phoQ deletion results in poor survival of this bacterium in macrophages, a marked attenuation that has been used for targeted delivery of tumoricidal proteins [46, 47]. In this study, we used attenuated Salmonella phoP/phoQ strain as the vector to deliver the plasmid to the tumor and for intranasal drug delivery to observe the* in vivo *effects of ER*β* on orthotopic PCa.

Our previous studies have included the construction of the recombinant plasmid PCDNA3.1-ER*β* with the human estrogen receptor 2 (ESR2) full-length cDNA [23]; we have already studied the effects of ER*β* on proliferation, apoptosis, and invasion in transfected PC-3M cells (dates were not shown). Thus, the goal of the present study was to find out the* in vivo* effects of ER*β* in the hormone-independent prostate cancer cell line PC-3M, and the signaling mechanisms that participate in the antiapoptotic effect of ER*β*. In this study, we used the model of orthotopic PCa in mice. Our results showed that the levels of ER*β* were upregulated in cancer tissues after PQ-PCDNA3.1-ER*β* treatment, indicating that the attenuated Salmonella can deliver the PCDNA3.1-ER*β* plasmid successfully into cancer cells, result in the apoptosis process, and thereby elicit a better therapeutic effect. We found that the mice in the PQ-PCDNA3.1-ER*β* group were in a healthier state with a decreased degree of cachexia. Furthermore, the mean body weights of mice in this group were higher and the mean weights of tumor were notably lower, compared to those of the mice in the MOCK, PQ, and PQ-PCDNA3.1 groups (Table 1). This indicates the obvious antitumor effect of ER*β*. Because of the safety and efficacy of attenuated Salmonella carrying the plasmid, we did not show the details of the analysis of bacterial distribution. We then performed Annexin V-FITC and TUNEL assays to see the effect of ER*β* on apoptosis. The results showed that the number of apoptotic cells in the PQ-PCDNA3.1-ER*β* group was significantly increased (Figures 1 and 2(b)). PCNA is a nuclear proliferation antigen and its activation is closely related to cell proliferation [48]. Next, we performed PCNA staining to detect the effect of ER*β* on cell proliferation. Our immunohistochemical examination showed that the number of PCNA-positive cells was lower in the PQ-PCDNA3.1-ER*β* treatment group than in the MOCK, PQ, and PQ-PCDNA3.1 groups (Figure 2(a)), indicating that the downregulated expression of PCNA may be due to the increased expression of ER*β*, leading to the inhibition of the proliferation of cancer cells. This is consistent with the results of the study by Bardin et al. which suggested that the protective role of ER*β* was based on direct (ER*β*-specific) effects limiting cell proliferation [49], and also the studies of Jarred et al., which suggested that activating ER*β* reduced proliferation* in vitro* in cell lines and also reduced the development of PCa in animal models [50]. These findings reveal that the attenuated Salmonella carrying the PCDNA3.1-ER*β* plasmid can exert a potent antitumor effect* in vivo* by suppressing proliferation and promoting the apoptosis of the cancer cells.

To further clarify the mechanisms by which ER*β* induces apoptosis, we analyzed the expression of ER*β* and apoptosis-related proteins by western blot. We first compared the expression levels of ER*β* and apoptosis-related proteins between the MOCK and PQ groups to detect the effect of attenuated Salmonella on tumors; the results showed that there were no obvious changes in the expression levels of these proteins after attenuated Salmonella treatment (Figure 3). We then compared the results of the PQ-PCDNA3.1 and PQ-PCDNA3.1-ER*β* groups. We found that the expression of ER*β* increased in the PQ-PCDNA3.1-ER*β* group compared with the PQ-PCDNA3.1 group (Figure 4), because transfecting the full-length ER*β* gene increased ER*β* expression in the PCDNA3.1-ER*β* plasmid. Akt is activated in response to many extracellular stimuli like insulin-like growth factor, nerve growth factor, and so on [51, 52]; it can impede the normal apoptotic response by suppressing the activity of numerous proapoptotic proteins, including the downstream target gene Bad and caspase 9 which mediates apoptosis [53, 54]. Thus, we assessed the effect of ER*β* expression on Akt signaling. The results showed that, compared to the PQ-PCDNA3.1 group, the expression of Akt and phosphorylated Akt (p-Akt) decreased in the PQ-PCDNA3.1-ER*β* group (Figure 4). This is consistent with the results of Lindberg et al., who conducted studies on T47-D ER*β* and MCF-7 ER*β* cells and found that the expression of ER*β* clearly downregulated the expression of phosphorylated Akt (p-Akt) [55]. We also found that the decreased Akt activity corresponds to the enhanced expression of the proapoptotic protein Bad. This is consistent with the results of the studies by Jun et al. [56]. Additionally, studies by Al-Bazz et al. found a significant correlation between the expressions of Akt and Bad [57]. Bad promotes cell death by interacting with antiapoptotic Bcl-2 members such as Bcl-xl [58, 59]. Our results showed that the ratio of Bad/Bcl-xl was elevated in the PQ-PCDNA3.1-ER*β* group (Figure 4). In addition, since the ratios of proapoptotic proteins (e.g., Bad and Bax) and antiapoptotic proteins (e.g., Bcl-2 and Bcl-xl) are essential for the regulation of apoptosis through caspase signaling, the increased ratios of Bad/Bcl-xl could initiate the caspase activation pathway for apoptosis [60]. Caspase 9 is a critical initiator caspase, and caspase 3 is a terminator caspase, and both are implicated in the execution of apoptosis, which lead to DNase activation followed by DNA fragmentation [61, 62]. The results in the PQ-PCDNA3.1-ER*β* group showed that the p-caspase 9 and p-caspase 3 expressions were increased accompanied by an increased cleavage of PARP, which ultimately lead to apoptosis (Figure 4). Chen et al. showed that ER*β* triggers apoptosis notably by increasing the levels of p-caspase 3 and cleavage of PARP [63]. This was also confirmed by our results. Collectively, our studies have demonstrated that ER*β* can upregulate the expression of several proapoptotic proteins such as Bad, activated caspase 9, and activated caspase 3 and downregulate the expression of the antiapoptotic proteins Akt and Bcl-xl, which are key components of the apoptosis pathway.

In summary, we showed that ER*β* could inhibit tumor cell proliferation and induce apoptosis. These effects are because the increased expression of ER*β* could influence the expression of Akt and its downstream target genes, thereby resulting in the induction of apoptosis. From these data, we can conclude that an ER*β* mediated signaling can affect the progression of PCa carcinogenesis in human prostate tissues. Thus, a better understanding of ER*β* expression, which is regulated throughout the natural history of the disease, may yield new strategies for the diagnosis, prevention, and treatment of PCa.

## Figures and Tables

**Table 1:** Comparisons of body weight and tumor weight of mice from the various groups (mean ± SE).

Group (*n* = 8)	Mean weight of the mice (g)	Mean weight of the tumor (g)
MOCK	14 ± 1.4142	0.5155 ± 0.1165
PQ	15.5 ± 1.8708	0.4886 ± 0.1589
PQ-PCDNA3.1	14.83 ± 0.7527	0.5140 ± 0.1454
PQ-PCDNA3.1-ER*β*	19.5 ± 2.0736^*∗*^	0.2424 ± 0.0324^*∗*^

^*∗*^ *P* < 0.05, versus the MOCK, PQ, and PQ-PCDNA3.1 groups.

## Data Availability

The research article data used to support the findings of this study are included within the article.
